# Utility of a transabdominal ultrasound-guided method with digital rectal examination for difficult urethral catheterization

**DOI:** 10.1186/2036-7902-4-S1-A9

**Published:** 2012-12-18

**Authors:** Toru Kameda, M Fujita, A Isaka, M Ozawa

**Affiliations:** 1 Department of Emergency Medicine, Red Cross Society Azumino Hospital, Azumino, Japan

## Objective

We evaluated the utility of a transabdominal ultrasound-guided method with digital rectal examination performed by emergency medical personnel for difficult male urethral catheterization.

## Methods

This study investigated male patients in whom standard urethral catheterization attempted by an emergency nurse or emergency physician failed in our institution or who were transferred from other hospitals or nursing homes following failure of the procedure and subsequent urethral bleeding. Patients with a history of urological surgery were excluded. Transabdominal ultrasonography was performed using a portable device with a 2-5 MHz convex probe. First, an emergency physician placed the probe on the suprapubic region longitudinally and observed the possible course of the prostatic to bulbar urethra, and tried to detect the tip of a catheter advanced by a nurse until progress was obstructed. To detect the tip more easily, the physician asked the nurse to oscillate the catheter and moved the tip when necessary. After the tip was detected, the nurse withdrew 2-3 cm. The physician then inserted the index finger of the opposite side into the rectum and kept pushing the site of the previous resistance ventrally while simultaneously holding the probe. After following these procedures, the nurse advanced the catheter again.

## Results

Five patients (age range, 56-93 years) were enrolled between March 2011 and April 2012. The tip of the catheter was observed in the bulbomembranous urethra or the false passage with transabdominal ultrasonography in four of the five patients. In these four patients, the false passage was compressed or the curve of the bulbomembranous urethra became gentle by pushing the regions ventrally from the rectum, and the tip was advanced smoothly to the bladder.

## Conclusion

This transabdominal ultrasound-guided method with digital rectal examination performed by emergency medical personnel appears useful for overcoming difficult urethral catheterization in some male patients.

